# Improving appropriate polypharmacy for older people in primary care: selecting components of an evidence-based intervention to target prescribing and dispensing

**DOI:** 10.1186/s13012-015-0349-3

**Published:** 2015-11-16

**Authors:** Cathal A. Cadogan, Cristín Ryan, Jill J. Francis, Gerard J. Gormley, Peter Passmore, Ngaire Kerse, Carmel M. Hughes

**Affiliations:** School of Pharmacy, Queen’s University Belfast, 97 Lisburn Road, Belfast, BT9 7BL UK; School of Pharmacy, Royal College of Surgeons in Ireland, Dublin, Ireland; School of Health Sciences, City University London, London, UK; Department of General Practice, Queen’s University Belfast, Belfast, UK; Centre for Public Health, Queen’s University Belfast, Belfast, UK; School of Population Health, University of Auckland, Auckland, New Zealand

**Keywords:** Polypharmacy, Intervention, Qualitative, Behaviour change, Prescribing, Dispensing, Theoretical Domains Framework

## Abstract

**Background:**

The use of multiple medicines (polypharmacy) is increasingly common in older people. Ensuring that patients receive the most appropriate combinations of medications (appropriate polypharmacy) is a significant challenge. The quality of evidence to support the effectiveness of interventions to improve appropriate polypharmacy is low. Systematic identification of mediators of behaviour change, using the Theoretical Domains Framework (TDF), provides a theoretically robust evidence base to inform intervention design. This study aimed to (1) identify key theoretical domains that were perceived to influence the prescribing and dispensing of appropriate polypharmacy to older patients by general practitioners (GPs) and community pharmacists, and (2) map domains to associated behaviour change techniques (BCTs) to include as components of an intervention to improve appropriate polypharmacy in older people in primary care.

**Methods:**

Semi-structured interviews were conducted with members of each healthcare professional (HCP) group using tailored topic guides based on TDF version 1 (12 domains). Questions covering each domain explored HCPs’ perceptions of barriers and facilitators to ensuring the prescribing and dispensing of appropriate polypharmacy to older people. Interviews were audio-recorded and transcribed verbatim. Data analysis involved the framework method and content analysis. Key domains were identified and mapped to BCTs based on established methods and discussion within the research team.

**Results:**

Thirty HCPs were interviewed (15 GPs, 15 pharmacists). Eight key domains were identified, perceived to influence prescribing and dispensing of appropriate polypharmacy: ‘Skills’, ‘Beliefs about capabilities’, ‘Beliefs about consequences’, ‘Environmental context and resources’, ‘Memory, attention and decision processes’, ‘Social/professional role and identity’, ‘Social influences’ and ‘Behavioural regulation’. Following mapping, four BCTs were selected for inclusion in an intervention for GPs or pharmacists: ‘Action planning’, ‘Prompts/cues’, ‘Modelling or demonstrating of behaviour’ and ‘Salience of consequences’. An additional BCT (‘Social support or encouragement’) was selected for inclusion in a community pharmacy-based intervention in order to address barriers relating to interprofessional working that were encountered by pharmacists.

**Conclusions:**

Selected BCTs will be operationalised in a theory-based intervention to improve appropriate polypharmacy for older people, to be delivered in GP practice and community pharmacy settings. Future research will involve development and feasibility testing of this intervention.

**Electronic supplementary material:**

The online version of this article (doi:10.1186/s13012-015-0349-3) contains supplementary material, which is available to authorized users.

## Background

The use of multiple medicines, also termed polypharmacy, is increasingly common in older people [1, 2]. This has been attributed to several factors including the high prevalence of multimorbidity (i.e. the presence of two or more chronic conditions) in older populations and the large number of evidence-based guidelines which advocate the use of more than one drug in the management of long-term conditions, such as hypertension [3]. Ensuring that older people receive the most appropriate combinations of medications is an ongoing challenge faced by healthcare professionals (HCPs). Polypharmacy has been identified as the principal determinant of potentially inappropriate prescribing for older populations [4, 5] and linked to negative clinical consequences, including adverse drug events (ADEs), drug interactions and medication non-adherence [6].

Although there is no universally accepted definition of the term ‘polypharmacy’, much of the literature to date has been based around numerical thresholds, such as the co-prescribing of four or five medications [6]. Historically, polypharmacy has been viewed negatively and seen to signify inappropriate medication use [7]. However, recent cohort studies have challenged existing assumptions that polypharmacy is always hazardous or indicative of poor care and highlighted the need for greater consideration of the clinical context in which medications are prescribed [8, 9]. Accordingly, the term ‘appropriate polypharmacy’ has been promoted in place of existing thresholds that define polypharmacy based on the number of medications prescribed to patients [10]. Appropriate polypharmacy is defined as the ‘prescribing for individuals with complex or multiple conditions where medicine use has been optimised and prescribing aligns with best evidence’ [10]. The concept of ‘appropriate polypharmacy’ recognises that patients can benefit from multiple medications provided that prescribing is evidence-based and reflects patients’ clinical needs.

A recently updated Cochrane review highlighted a lack of rigorous evaluations of interventions to improve appropriate polypharmacy in older people [11]. Although the review findings supported a limited range of interventions, which were most commonly pharmaceutical care-based and typically involved medication reviews by HCPs, considerable limitations were identified with the existent literature (e.g. risks of bias, insufficient details of intervention development and delivery) [11]. In order to address these deficiencies, it was recommended that future research adopt a more systematic approach in developing interventions using a combination of evidence and theory [11].

The study reported here was part of a larger multiphase mixed methods research project that sought to address identified limitations of previous interventions targeting appropriate polypharmacy in older people. The specific aim of the project was to develop an intervention using a systematic approach that was guided by the best available evidence and appropriate theory and also engaged key stakeholders in the provision of medications to older patients in primary care (i.e. general practitioners (GPs) and community pharmacists). This approach to intervention development was modelled on the Medical Research Council’s (MRC) guidance for developing and evaluating complex interventions [12]. Linked components of the larger project involved the update of a Cochrane review [11], as noted above, in order to identify the current evidence base for interventions that aimed to improve appropriate polypharmacy. The objective of the current study was to identify the underlying barriers to, and enablers of, prescribing/dispensing appropriate polypharmacy and to frame these barriers and enablers in terms of theoretical domains of behaviour change to support intervention development. The underpinning theoretical model was the Theoretical Domains Framework (TDF) [13]. The TDF has been used to identify key theoretical domains that are perceived to influence HCPs’ behaviours and provides a theoretically robust evidence base to inform intervention design. Key domains are then mapped to behaviour change techniques (BCTs) which can form the intervention’s so-called 'active ingredients' [14]. Michie et al. [15] previously developed a matrix to aid this mapping process using the original 12 domain TDF (TDF1) [13]. Cane et al. [16] recently developed this work using the 14 domain TDF (TDF2) [17] and a larger number of BCTs.

A number of previous TDF-based intervention development studies have focussed on the implementation of evidence-based guidelines by HCPs as the target behaviour [18–20]. The challenge of targeting HCPs’ clinical behaviours to improve appropriate polypharmacy was a lack of evidence and guidelines to inform clinical practice when prescribing for older patients, in whom multimorbidity is highly prevalent [21]. This study used the TDF to focus specifically on the prescribing and dispensing of appropriate polypharmacy to older patients by GPs and community pharmacists, respectively, as the target behaviours. The study aimed to identify key theoretical domains that may act as mediators (i.e. barriers, facilitators) of behaviour change and to map these domains to BCTs that could be incorporated into an intervention to improve appropriate polypharmacy in older people in primary care. The identified intervention components (i.e. BCTs) were used to develop an evidence-based and well-theorised intervention (details of which will be reported in a future publication) ready for feasibility testing at a later stage of the project, before progressing to a larger scale evaluation in a randomised study.

## Methods

Semi-structured interviews were conducted with GPs and community pharmacists using separate TDF-based topic guides (Additional file 1 and Additional file 2) to identify key theoretical domains which were then mapped to BCTs that would be the components of an intervention to improve appropriate polypharmacy in older patients. Ethical approval was granted by the Office of Research Ethics Committees Northern Ireland (REC reference 13/NI/0114).

### Sampling and recruitment

A purposive sampling method was employed. A computer-generated random sample of general practices from each of five Health and Social Care Trust areas (main administrative health areas) in Northern Ireland was contacted by telephone by a nurse practitioner from the Northern Ireland Clinical Research Network (NICRN). The nurse provided a brief summary of the project and posted a written invitation letter and information sheet to practices that expressed interest in receiving further information. The nurse aimed to recruit up to two GPs from two general practices (one urban, one rural) in each Health and Social Care Trust area to ensure geographical diversity. The nurse liaised with each practice to arrange dates for the researcher (CC) to attend the practice to interview GP participants.

Practice managers in recruited practices were asked to identify local community pharmacies that dispensed most of the practice’s prescriptions for older patients. These pharmacies were contacted by telephone by the researcher who provided a brief summary of the project and posted a written invitation letter and information sheet to those interested in receiving further information. One community pharmacist from up to two pharmacies associated with each participating general practice was invited to participate. The researcher arranged interviews with pharmacist participants.

The researcher obtained written informed consent from all participants prior to taking part in the research. All participants were offered financial compensation for their time (£50 cheque) and provided with a certificate of participation for their continuing professional development portfolios.

### Semi-structured interviews

Semi-structured face-to-face interviews were conducted by the researcher (CC) with recruited HCPs either at their place of work or another convenient location. Interviews were conducted between May and October 2014. Interview topic guides were based around the 12 domains of the TDF [13] and developed through discussion amongst members of the research team. The 12-domain version of the TDF was specifically chosen for a number of reasons. Based on preliminary discussions within the research team, as well as individual team members’ (CR, JF) previous experience in using TDF1 [18, 19, 22], it was agreed that the ‘Nature of the behaviours’ domain would likely be important to the target behaviours. For example, it was felt that the extent to which prescribing and dispensing of appropriate polypharmacy formed part of routine practices would need to be explored using this domain which is present in TDF1 but not in TDF2 (14 domain). In addition, we wanted to avoid unnecessarily long interviews in order to minimise the burden to participants and considered TDF1 to be more parsimonious than TDF2. We were also aware of some evidence in the literature that TDF1 does not have poorer validity than TDF2 [23].

Separate interview topic guides were developed and piloted for each HCP group. Each topic guide comprised a similar line of questions covering four key areas: (1) HCPs’ views on the term ‘polypharmacy’, (2) HCPs’ assessment of a clinical scenario depicting an older patient receiving inappropriate polypharmacy, (3) HCPs’ perceptions of barriers and facilitators to ensuring the prescribing (GPs) and dispensing (community pharmacists) of appropriate polypharmacy to older people and (4) HCPs’ views on potential intervention components and outcome measures for inclusion in future intervention studies. Participants’ assessments of the clinical scenario were not included in the analysis as the scenario was intended purely as a discussion aid to ensure that participants understood the nature of the target behaviours. Hence, participants were not prompted to provide a detailed assessment of the scenario during interviews. For other questions, prompts were used to encourage participants to elaborate on their responses where necessary.

Data were audio-recorded and transcribed verbatim. Transcripts were independently checked for accuracy, de-identified and assigned anonymous codes to indicate whether participants were GPs (i.e. GP01, GP02, etc.) or pharmacists (i.e. PCT01, PCT02, etc.).

### Data analysis

Each interview was analysed separately, and all transcripts were analysed independently by at least two members of the research team. An extensive familiarisation process was carried out which involved reading and re-reading of transcripts, as well as listening back to interview recordings. Data were analysed in two stages. The first stage used the framework method [24] and adopted a deductive approach using pre-defined coding categories: (1) healthcare professionals’ definitions of polypharmacy, (2) TDF domains (outlined further below), and (3) potential intervention components and assessment outcomes. The second stage used content analysis [25] and adopted an inductive approach in which emerging content themes relating to barriers and facilitators within each domain were identified. Transcript coding was compared and any disagreements were resolved by consensus discussion. NVivo QSR 10 was used to manage the data. As the primary focus of the analysis was the TDF-related data, the analysis of other sections of interview transcripts is not reported on in this paper.

#### Identification of key theoretical domains

The framework method was used to systematically index and chart the data using the 12 domains of the TDF as the coding categories (Additional file 3) [24]. Data were indexed by two researchers working independently. Indexed data were charted by one researcher in a Microsoft Excel spreadsheet to generate a framework matrix which included references to illustrative quotes.

Content analysis of the framework matrix was performed, and a summary outlining subthemes/specific beliefs within each domain was reviewed by other members of the research team. This summary was discussed by the research team who first identified criteria for deciding the relevance of each domain to the target behaviours (i.e. prescribing and dispensing of appropriate polypharmacy). Decisions about the relevance of each domain were guided by previous research [25]; the extent to which sections of interview transcripts were coded to each domain was reviewed as a crude indicator of relevance, and the summary documents were then used to determine whether participants related the domain to the target behaviour. Key theoretical domains for the intervention were selected by the multidisciplinary research team using a consensus-based approach. Decisions were based on barriers and facilitators within relevant domains that could feasibly be targeted based on the available project resources.

#### Triangulation

Two kinds of triangulation were conducted: data triangulation (use of multiple groups of research participants) and investigator triangulation (use of multiple researchers in data analysis) [26]. Triangulation methods focussed on comparing and contrasting both HCP groups’ perceptions of barriers and facilitators within each of the theoretical domains with a view to guiding the research team’s decisions as to how selected BCTs could eventually be operationalised as part of an intervention.

### Identification and selection of BCTs

A table of BCTs that have been reliably allocated to the 14 domain version of the TDF [17] by a panel of behaviour change experts was used as the primary reference source to guide the identification and selection of BCTs to target key theoretical domains [16]. The reason for using this mapping work as the primary reference sources was because it included a larger number of BCTs than the original mapping matrix that was developed by Michie et al. [15]. In order to inform identification and selection of BCTs that had not been allocated by Cane et al. [16] to domains such as ‘Social/professional role and identity’, the research team compared the results of this mapping process against the original mapping matrix [15] as a secondary reference source. The research team reviewed all of the BCTs that had been mapped to key domains in each reference source and reached consensus as to which BCTs should be selected for inclusion in the intervention. This selection process was guided by the interview data. For example, if time and workload were reported by HCPs as major barriers to the target behaviours, BCTs that could likely be delivered as part of a single intervention were to be prioritised over those that would require repeated administration or extended time periods to elicit required changes. This was to ensure that the intervention would be practicable for HCPs working in clinical practice.

## Results

### Participants

Eighty general practices and 25 community pharmacies were contacted about the research. Thirty HCP participants (15 GPs, 15 community pharmacists) were recruited from nine general practices (response rate 11 %) and 15 community pharmacies (response rate 60 %) across each of the five Health and Social Care Trust areas in Northern Ireland (Table 1). Despite repeated efforts by NICRN, it was not possible to recruit a second general practice within one of the trust areas into the study. Data saturation was reached after 13 GP interviews and 14 community pharmacist interviews. The additional interviews with each HCP group were conducted before a more detailed analysis was completed, and the point of data saturation was established. The duration of interviews varied amongst GP (range 32–84 min) and pharmacist (range 26–101 min) participants.

**Table 1:** Participant demographics

	General practices^a^ (*n* = 9)	Community pharmacies (*n* = 15)
Participant gender		
Male	10	7
Female	5	8
Years of professional experience (range)	3–27	3–32
Healthcare trust area		
1	4	4
2	1	2
3	3	3
4	3	2
5	4	4

^a^>1 GP participant was recruited from five general practices

### Summary of findings from analysis at the level of theoretical domains

The factors within each of the domains that were perceived to influence the prescribing and dispensing of appropriate polypharmacy to older people are summarised below.

Clinical experience (‘Nature of the behaviours’) was a key factor that facilitated HCPs in ensuring that older patients received appropriate polypharmacy. This was perceived to have an important influence on a number of other domains. For example, HCPs frequently referred to the role of clinical experience in developing their clinical knowledge (‘Knowledge’), competencies (‘Skills’) and professional confidence (‘Beliefs about capabilities’), all of which helped them to ensure that older patients received appropriate polypharmacy.

*“…experience is the thing that teaches you the most because it’s the thing that you tend to remember…” PCT2*

*“I think the confidence comes from experience.” GP2*

Both groups of HCPs reported that ensuring that older patients received appropriate polypharmacy was part of their professional role (‘Social/professional role and identity’) and they were aware of the potential for adverse outcomes (e.g. side-effects, drug interactions, non-adherence) if actions were not taken to improve appropriate polypharmacy in this patient cohort (‘Beliefs about consequences’).

*“…ultimately the buck stops with me.” GP2*

*“I know it’s easier to just go, ‘Oh should I just give it out or whatever’, but… you’re the one giving it out, I think you do have responsibility to, if you think something’s not appropriate, to double check.” PCT1*

Routine procedures within daily practice enabled HCPs to ensure that patients received appropriate polypharmacy (‘Nature of the behaviours’). These included monitoring and reviewing patients’ medication usage on an ongoing basis. However, for a number of HCPs, their levels of attention (‘Memory, attention and decision processes’) and commitment (‘Motivation and goals’) to carrying out these procedures as part of routine practice were lacking, particularly in light of existing time and workload barriers (‘Environmental context and resources’).

*“…it's possible that things are overlooked in a busy day to day practice.” GP9*

*“If somebody comes in about four other things, and you’ve got 10 minutes to see them in– it probably becomes, honestly probably becomes less of a priority… I think it’s not a fixed priority.” GP14*

*“…there's no money involved in addressing polypharmacy, you know, you could be completely cynical and turn round and go, ‘This patient gets 12 items, I get paid 98p for each item I dispense. Why do I want to go out and tell this man he only needs seven of them?’” PCT2*

Pharmacists were mindful of professional boundaries with GPs in recommending changes to older patients’ existing prescriptions (‘Social/professional role and identity’), and some doubted their ability to have recommended medication changes implemented by GPs (‘Beliefs about capabilities’). In contrast, GPs viewed teamwork with pharmacists favourably (‘Social influences’/‘Social/professional role and identity’). Based on their experiences with local community pharmacists or having had dedicated practice pharmacists to review prescribing practices, GPs considered pharmacists to be both an important resource (‘Environmental context and resources’) and an integral component of any strategy to improve appropriate polypharmacy in older people (‘Behavioural regulation’).

*“…pharmacists seem to be very well clued into it all already, you know, and I think they’re probably going to be main kind of stakeholders as well in reducing polypharmacy…” GP4*

*“…it doesn’t necessarily have to be a doctor doing it, and em, I think sometimes the pharmacists look at things in a different way than we do… it’s a different kind of focus you have on things…” GP14*

*“I think very often GPs eh or GP surgeries don’t often, value the intervention, interaction and, that is frustrating, continues to be frustrating, and I don’t see any improvement in it in the last 25 years of working. Don’t see any improvement in it at all, in fact I would say quite probably the reverse…” PCT10*

GPs outlined challenges in terms of professional boundaries with hospital prescribers and some GPs were reportedly unwilling to challenge recommendations from secondary care (‘Social/professional role and identity’).

*“…there are few GPs who will challenge the recommendations of a specialist and if they say, ‘Add in this, add in that’, they will often get added.” GP11*

Both HCP groups reported a number of groups that had variable influences on their clinical behaviour (‘Social influences’). The specific influence of patients and other HCPs varied according to the individual participant. GP receptionists were perceived as a barrier to ensuring that older patients received appropriate polypharmacy owing to difficulties encountered by pharmacists in making direct contact with GPs.

*“I mean another barrier I suppose is sometimes due to the receptionists in surgeries as well. Getting them to buy into the fact that clinical decisions should be taken between healthcare professionals, like pharmacists, nurses, doctors and not taken by receptionists which I find is happening a lot.” PCT15*

In a similar manner, GPs reported the influence of prescribing advisors as a barrier to ensuring that patients received appropriate polypharmacy.

*“…the barriers that come up to us for polypharmacy is, em, a lot of it is created from our prescribing advisors, to try and get us to get patients off, you know, medications and switch to other appropriate medications.” GP2*

Both HCP groups reported that they had the necessary clinical knowledge (‘Knowledge’) and skills/competencies (‘Skills’) to review older patients’ medications. This enabled them to ensure that patients received appropriate polypharmacy. However, some GPs discussed the challenges of applying available evidence and guideline recommendations in the context of older patients (‘Knowledge’/‘Skills’).

*“There might be evidence for each individual drug but, you know, I don’t think there’s– there’s no randomised controlled trials on, you know, what all those drugs in combination do and, you know, there aren’t going to be.” GP1*

*“Guidelines tend to be very disease-specific but how do you do something? How do you prescribe for somebody who has comorbidities and the guideline would seem to be talking against each other, and so we need the– where is the evidence base for telling people to do then?” GP8*

The main barriers that hindered both HCP groups in ensuring that older patients received appropriate polypharmacy related to the current work environment (‘Environmental context and resources’). For example, in addition to time and workload barriers, a lack of appropriate resources (e.g. staff) was also noted by both groups. Pharmacists raised other challenges that they encountered in practice (e.g. lack of direct communication network with GPs, lack of access to patients’ clinical data, lack of availability of non-pharmacological treatment options).

*“…it’s a mountain of work and the reason why it’s not being done is because it’s not being resourced and there’s no money to do it.” GP11*

As previously noted, barriers identified under the ‘Environmental context and resources’ domain impacted negatively on other domains (e.g. Memory, attention and decision processes, Motivation and goals). Various resources and practical strategies were outlined by both groups that either helped them in current practice (e.g. computer support systems) or would be helpful in the future (e.g. additional support staff). Limitations of existing computer support systems and information resources [e.g. British National Formulary (BNF), a standard reference text] were noted, particularly in terms of their clinical relevance and application in the context of older patients receiving polypharmacy.

*“....quite often the computer's flagging up ones that there isn't really an interaction.” PCT5*

*“…sometimes what’s in the BNF is not really enough. It does not match having a detailed past experience of prescribing something.” GP 1*

‘Emotion’ was the least frequently discussed domain and was not perceived to have an important influence on either group’s clinical behaviour.

### Identification of key domains

Based on the research team’s review of the summary findings from each dataset, all of the domains except for ‘Emotion’ were considered to be relevant to the prescribing and dispensing of appropriate polypharmacy to older patients. Using the methods previously described, eight key domains were selected to be targeted as part of an intervention involving GPs and/or community pharmacists: ‘Skills’, ‘Beliefs about capabilities’, ‘Beliefs about consequences’, ‘Environmental context and resources’, ‘Memory, attention and decision processes’, ‘Social/professional role and identity’, ‘Social influences’ and ‘Behavioural regulation’.

### Mapping of theoretical domains to BCTs

Using previous work on mapping BCTs to the TDF [15, 16], as already outlined, the research team identified and selected four BCTs for inclusion in a future intervention involving GPs and/or community pharmacists to improve appropriate polypharmacy in older people: ‘Action planning’, ‘Prompts/cues’, ‘Modelling or demonstrating of behaviour’ and ‘Salience of consequences’.

‘Social support or encouragement’ was selected as an additional BCT for inclusion in the community pharmacy-based intervention. This BCT mapped to both ‘Social/professional role and identity’ and ‘Social influences’ and was specifically included to target the professional boundary and team working-related issues that were identified in the community pharmacist interviews

Details of the mapping process used to select BCTs to target mediators identified within the key domains are provided in Table 2. It must be noted that the ‘Nature of the behaviours’ domain is distinct from the other theoretical domains as it relates to the key characteristics of the behaviour of interest as opposed to potential mediating mechanisms or influences [13, 27]. Hence, it was not included in the original BCT mapping exercise undertaken by Michie et al. [15], nor was it included in the mapping exercise that was conducted in the current project. It was intended that the habitual processes that were coded under this domain would be modified indirectly by targeting other causal aspects of the target behaviours (i.e. the other key domains) using selected BCTs [28].

**Table 2:** Mapping of behaviour change techniques (BCTs) to key domains for inclusion in an intervention to improve appropriate polypharmacy in older people

Domain	BCTs identified from Cane et al. 2015 [16]	Additional BCTs identified from mapping matrix [15]	Selected BCTs as proposed intervention components (including reasons to justify exclusion of other BCTs)
Skills	1. Graded tasks 2. Behavioural rehearsal/practice 3. Habit reversal 4. Body changes 5. Habit formation	6. Goal/target specified: behaviour or outcome 7. Monitoring 8. Self-monitoring 9. Rewards; incentives (inc Self-evaluation) 10. Graded task: start with easy tasks 11. Increasing skills: problem solving, decision making, goal setting 12. Rehearsal of relevant skills 13. Modelling/demonstration of behaviour by others 14. Homework 15. Perform behaviour in different settings	Modelling/demonstration of behaviour by others (BCT 13): HCPs would be provided with a demonstration of how to prescribe/dispense appropriate polypharmacy during a typical encounter/consultation with an older patient.
			Reasons for not selecting other BCTs
			BCTs 1, 2, 3, 5, 7, 8, 10, 12, 14: likely to require repeated administration and/or extended time periods to effect required changes in target behaviours.
			BCT 4: not applicable as a direct change to HCPs’ body structure/functioning is unlikely to have an impact on the target behaviours (i.e. prescribing/dispensing of appropriate polypharmacy).
			BCT 6: not possible to establish an acceptable goal/target in terms of the number of older patients that HCPs would perform target behaviours on because ideally the target behaviours should be performed on all older patients.
			BCT 9: not within scope of project to offer rewards/incentives and a general practice-based incentive scheme already exists in UK (i.e. the Quality and Oucomes Framework).
			BCT 11: intervention would likely need to be tailored to individual HCPs to account for baseline variation in skill levels.
			BCT 15: not applicable as the intervention will target HCPs in their normal place of work (i.e. general practice, community pharmacy) and the intervention will focus on the prescribing/dispensing of appropriate polypharmacy to community dwelling older patients as opposed to patients in other settings (e.g. nursing homes) whose clinical complexity and context is likely to be very different.
Beliefs about capabilities	1. Verbal persuasion to boost self-efficacy 2. Focus on past success	3. Self-monitoring 4. Graded task: start with easy tasks 5. Increasing skills: problem solving, decision making, goal setting 6. Coping skills 7. Rehearsal of relevant skills 8. Social processes of encouragement, pressure, support 9. Modelling/demonstration of behaviour by others 10. Homework 11. Perform behaviour in different settings	Social processes of encouragement, pressure, support (BCT 8): mapped to ‘Social/professional role and identity’ and ‘Social influences' – see below.
			Modelling/demonstration of behaviour by others (BCT 9): outlined above – see ‘Skills’ domain.
			Reasons for not selecting other BCTs
			BCTs 3, 4, 10: likely to require repeated administration and/or extended time periods to effect required changes in target behaviours.
			BCT 1: intervention would likely need to be tailored to individual HCPs to account for baseline variation in self-efficacy levels.
			BCT 2: not suitable due to potential variation in experience amongst HCPs (i.e. if HCPs do not have previous experience of performing the target behaviours then this BCT will not apply to them).
			BCTs 5, 6, 7: intervention would likely need to be tailored to individual HCPs to account for baseline variation in skill levels.
			BCT 11: not applicable as the intervention will target HCPs in their normal place of work (i.e. general practice, community pharmacy) and the intervention will focus on the prescribing/dispensing of appropriate polypharmacy to community dwelling older patients as opposed to patients in other settings (e.g. nursing homes) whose clinical complexity and context is likely to be very different.
Beliefs about consequences	1. Emotional consequences 2. Salience of consequences 3. Covert sensitization 4. Anticipated regret 5. Social and environmental consequences 6. Comparative imagining of future outcomes 7. Vicarious reinforcement 8. Threat 9. Pros and cons 10. Covert conditioning	11. Self-monitoring 12. Persuasive communication 13. Information regarding behaviour,outcome 14. Feedback	Information regarding behaviour, outcome/salience of consequences (BCTs 2 and 13 - equivalent): as part of the demonstration of how to prescribe/dispense appropriate polypharmacy during a typical encounter/consultation with an older patient, positive feedback would be included from the HCPs and patients to emphasise the positive consequences of performing the behaviour.
			Reasons for not selecting other BCTs
			BCTs 7, 10, 11, 14: likely to require repeated administration and/or extended time periods to effect required changes in target behaviours.
			BCT 1: emotional consequences of performing the target behaviours have not been established.
			BCTs 3, 4: not applicable as intervention is focussed on wanted behaviours as opposed to unwanted behaviours.
			BCT 5: not applicable as HCPs were already aware of the consequences of performing the target behaviours.
			BCT 6: intervention would likely need to be tailored to individual HCPs as the imagining and comparing of future outcomes of changed versus unchanged behaviour is likely to vary between individuals.
			BCT 8: not within scope of project to implement future punishment or removal of reward as a consequence of HCPs performing an unwanted behaviour.
			BCT 9: intervention would likely need to be tailored to individual HCPs because if advised to identify and compare pros and cons of performing the target behaviours, assessments are likely to vary between individuals.
			BCT 12: difficult to have a credible source present evidence-based arguments in favour of the target behaviours as few interventions to date have examined clinically relevant outcomes (e.g. hospital admissions, ADEs) and where these outcomes have been evaluated, the findings have been inconsistent.
Environmental context and resources	1. Restructuring the physical environment 2. Discriminative (learned) cue 3. Prompts/cues 4. Restructuring the social environment 5. Avoidance/changing exposure to cues for the behaviour	6. Environmental changes (e.g. objects to facilitate behaviour)	Prompts/cues (BCT 3): HCPs will be asked to arrange for support staff (e.g. receptionists, pharmacy technicians) to prompt them to check that older patients who meet specific criteria are prescribed/dispensed appropriate polypharmacy when patients present in the practice/pharmacy.
			Reasons for not selecting other BCTs
			BCT 1: not within scope of project to restructure HCPs’ physical work environment.
			BCTs 2: not within scope of project to offer reward (e.g. monetary fee) for performing target behaviour).
			BCT 4: not within scope of project to restructure HCPs’ social environment.
			BCT 5: not applicable as intervention is seeking to promote performance of target behaviours by HCPs as opposed to avoiding/reducing exposure to cues for the target behaviours.
			BCT 6: not within scope of project to restructure HCPs’ physical work environment.
Memory, attention and decision processes	None	1. Self-monitoring 2. Planning, implementation 3. Prompts, triggers, cues	Planning, implementation (BCT 2; equivalent to Action planning): HCPs would be asked to write an explicit plan of when and how they would ensure that patients meeting inclusion criteria are prescribed/dispensed appropriate polypharmacy.
			Prompts, triggers, cues (BCT 3): outlined under ‘Environmental context and resources’ domain.
			Reasons for not selecting other BCTs
			BCT 1: likely to require repeated administration and/or extended time periods to effect required changes in target behaviours.
Social influences	1. Social comparison 2. Social support or encouragement (general) 3. Information about others' approval 4. Social support (emotional) 5. Social support (practical) 6. Vicarious reinforcement 7. Restructuring the social environment 8. Modelling or demonstrating the behaviour 9. Identification of self as role model 10. Social reward	11. Social processes of encouragement, pressure, support 12. Modelling/demonstration of behaviour by others	Social processes of encouragement, pressure, support/ Social support or encouragement (BCT 2, 11 - equivalent): Pharmacists would receive a list of pre-approved patients from the GP practice which would encourage/support them in engaging with patients to ensure that they are dispensed appropriate polypharmacy.
			Modelling /demonstration of behaviour by others/ Modelling or demonstrating the behaviour (BCT 8, 12 - equivalent): outlined above – see ‘Skills’ domain.
			Reasons for not selecting other BCTs
			BCTs 6, 9, 10: likely to require repeated administration and/or extended time periods to effect required changes in target behaviours.
			BCT 1: difficult to draw meaningful comparisons between HCPs’ performance of target behaviours.
			BCT 3: difficult to establish older patients’ views on HCPs performing target behaviours due to clinical heterogeneity amongst older patients who are receiving polypharmacy in terms of clinical conditions and medication types.
			BCTs 4, 5: encapsulated by BCT 2.
			BCT 7: not within scope of project to restructure HCPs’ social environment.
Behavioural regulation	1. Self-monitoring of behaviour	2. Goal/target specified: behaviouror outcome 3. Contract 4. Planning, implementation 5. Prompts, triggers, cues 6. Use of imagery	Planning, implementation (BCT 4; equivalent to Action planning): see under ‘Memory, attention and decision processes’ domain above.
			Prompts, triggers, cues (BCT 5): see under ‘Environmental context and resources’ domain above.
			Reasons for not selecting other BCTs
			BCT 1: likely to require repeated administration and/or extended time periods to effect required changes in target behaviours.
			BCT 2: not possible to establish an acceptable goal/target in terms of the number of older patients that HCPs would perform target behaviours on because, ideally, the target behaviours should be performed on all older patients.
			BCT 3: not within scope of current project to impose additional contractual obligations on HCPs.
			BCT 6: used in the context of implementing other BCTs through the use of planned images (visual, motor, sensory); not applicable in the context of this research project.
Social/professional role and identity	None	1. Social processes of encouragement, pressure, support	Social processes of encouragement, pressure, support (BCT 1): see under ‘Social influences’ domain above.
Nature of the behaviours	No BCTs were mapped to this domain because it was not included in the original BCT mapping matrix [15]. It was intended that this domain would be targeted indirectly using the selected BCTs that were mapped to the other seven key domains.		

## Discussion

This study forms part of a systematic approach to develop an intervention to improve appropriate polypharmacy in older people in primary care that was modelled on the MRC framework [12]. This type of approach has been found to be lacking in the related literature [11]. The study advances application of the MRC framework in the intervention development phase as initial exploratory work was undertaken with GPs and community pharmacists as two groups of stakeholders that play key roles in the provision of medication to older people in primary care. The TDF [13] was used as an underpinning theoretical model to gather comprehensive insights into the clinical behaviours that need to be targeted in order to achieve appropriate polypharmacy. This provides the foundation for developing a theory- and evidence-based intervention using specific behaviour change techniques (BCTs) to target key mediators (i.e. barriers, facilitators) of behaviour change.

With the exception of the ‘Emotion’ domain, all of the theoretical domains were considered relevant to the target behaviours (i.e. prescribing and dispensing of appropriate polypharmacy). This illustrates the complex nature of the target behaviours, as well as the challenge faced by researchers in identifying key domains to target when developing interventions to change these behaviours. In selecting key domains, we noted that barriers within a number of domains could not realistically be addressed as part of the current project, such as the lack of available evidence and guidelines to support prescribing for older people with multimorbidity (i.e. barriers within the ‘Knowledge’ domain). Thus, we purposefully selected domains that would assist HCPs in taking action to ensure that patients were prescribed/dispensed appropriate polypharmacy (e.g. ‘Skills’, ‘Beliefs about capabilities’) as part of routine clinical practice (‘Nature of the behaviours’). The importance of the same group of key domains for both HCP groups highlights commonalities in the perceived mediators of behaviour change within each group. For example, the sense of professional responsibility (‘Social/professional role and identity’) expressed by members of both professions in ensuring that patients received appropriate polypharmacy facilitated HCPs in taking action to instigate medication changes where necessary. However, it must be noted that despite identification of similar challenges within a number of domains (e.g. time and work environment pressures under the ‘Environmental context and resource’ domain), perceptions of other domains as a barrier or facilitator differed between the groups. For example, each group’s perceptions differed as to the existence of interprofessional boundaries within primary care (‘Social/professional role and identity’).

Due to the selection of the same key domains, an overlap in the BCTs that would form the components of an intervention involving GPs and/or community pharmacists was expected (Table 2). Given the identified challenges relating to the current work environment (e.g. time and resource limitations), as well as the lack of available evidence comparing the effectiveness of BCTs in targeting specific theoretical domains, we prioritised BCTs that were unlikely to need repeated administration/delivery on a large number of occasions or over extended time periods to elicit required changes in the target group’s behaviour. In this way, ‘Modelling/demonstration of behaviour by others’ was chosen over other BCTs, such as ‘Graded tasks’, to target the ‘Skills’ domain. We also purposefully kept the number of BCTs to a minimum in order to ensure that any proposed intervention would be feasible to deliver to HCPs with high fidelity. We felt that this would be preferable on account of both the existing time constraints within HCPs’ current work environment and the lack of available evidence to support multifaceted interventions (i.e. an intervention including two or more components) over single-component interventions in changing HCPs’ behaviours [29]. A future paper will outline different ways to operationalise the selected BCTs to design the intervention.

This study has raised two important methodological issues regarding the development of TDF-based interventions targeting HCPs’ clinical behaviours. Firstly, we based our interpretation of the domains on definitions that were used in previous studies [22, 30]. Thus, in our interpretation of the ‘Nature of the behaviours’ domain, we coded references to ‘clinical experience’ in the interview transcripts to this domain because ‘Direct experience/past behaviour’ is listed as a theoretical construct under this domain. However, other authors have published additional definitions for each of the 14 theoretical domains of the most recent version of the TDF [16], as well as a separate definition of the ‘Nature of the behaviours’ domain [31]. Despite the intended purpose of the TDF to simplify psychological theory and make it more accessible to researchers [13], the lack of a single reference source of agreed definitions creates challenges in operationalising the TDF [32]. A single reference source with domain definitions, as well as illustrative examples of how domains can be operationalised during data collection and analysis, would be invaluable to researchers to ensure consistency in the interpretation and application of the TDF across studies. It would be important for any future reference source to include both versions of the TDF as the 12 domain version is still in use [19, 33, 34], and ongoing research suggests that it may be more applicable than the 14 domain version in developing a generic TDF-based questionnaire [23].

Secondly, our study highlights the challenge faced by researchers in selecting BCTs to target particular theoretical domains where there is a lack of agreement or guidance in the literature. Although the mapping work by Cane et al. [16] provided up-to-date guidance for most of the mapping process, it did not help us in selecting BCTs to target two key domains: ‘Memory, attention and decision processes’ and ‘Social/professional role and identity’. We sought to overcome this limitation by comparing the results of the mapping process against the original mapping matrix that was developed by Michie et al. [15]. It must be noted that there is, at present, no single best approach to map BCTs to theoretical domains. Given the lack of consensus amongst experts in the field in mapping BCTs to particular theoretical domains [15, 16], it is only after suitably designed experimental studies are conducted that researchers will have a clearer understanding as to which BCTs are effective in targeting specific domains. In the interim, we contend that the use of both reference sources provides a useful strategy to guide BCT selection.

### Strengths and limitations

In reporting the systematic approach that we used to identify key theoretical domains and to select BCTs as part of our intervention development programme, we have followed existing recommendations by being explicit about our underlying reasons and rationale [18, 35]. This should facilitate other researchers and practitioners in understanding how the underpinning theoretical framework was used to guide intervention development.

In addition to identifying mediators of behaviour change to target as part of the intervention, the interviews also provided valuable information about the clinical context in which the behaviours are currently performed. This information will be used to inform selection of intervention approaches as part of the feasibility screening process that will be outlined in a future paper. For example, given the notable limitations of existing computer-based prescribing alerts (‘Environmental context and resources’), such as lack of clinical relevance, it is unlikely that a computer-based intervention approach will be pursued. Another strength of this study was the inclusion of a health psychologist (JF) with expert knowledge of the TDF as part of the research team. This helped to ensure effective use of the TDF (i.e. topic guide development, data analysis) and that domains were not interpreted superficially [27]. As a further step to help overcome the challenge in clarifying boundaries between domains, all data were analysed independently by at least two members of the research team. This added to the validity and reliability of the coding process.

Limitations must be noted with the research design and sampling approach. As a qualitative study, the findings are not readily generalisable to the wider population of HCPs. However, participants were sampled across Northern Ireland, and the spread of geographical locations enhances the transferability of the findings. It must also be noted that participants comprised a self-selected sample, and participation was incentivised. Another limitation is that the findings reflect participants’ perceptions/attributions of influences on their clinical behaviours as opposed to actual causes [27]. In addition, clinicians’ beliefs regarding appropriate clinical practices may not necessarily translate into action at the individual patient level [36].

## Conclusions

The study findings highlight the range of theoretical domains that were perceived to influence HCPs when prescribing and dispensing appropriate polypharmacy to older people in primary care and, hence, the complex nature of the target behaviours. There was a considerable overlap in terms of the key domains that were perceived to affect both HCP groups’ behaviours, and a number of common BCTs were selected for inclusion in a future intervention involving GPs and/or community pharmacists. Selected BCTs provide a foundation for developing a theory-based intervention to improve appropriate polypharmacy in older people in primary care. Future work will involve developing an intervention using selected BCTs for further feasibility testing before any larger scale trial evaluation is undertaken.

## Additional files

Additional file 1:
**General practitioner interview topic guide.** TDF-based topic guide that was used to explore the theoretical domains as barriers and facilitators to the prescribing of appropriate polypharmacy for older people.(DOC 62 kb) Additional file 2:
**Community pharmacist interview topic guide.** TDF-based topic guide that was used to explore the theoretical domains as barriers and facilitators to the dispensing of appropriate polypharmacy to older people. (DOC 58 kb) Additional file 3:
**Description of 12 theoretical domains from TDF** **[** 13 **]**. Descriptions of the theoretical domains that were used to code the interview data. (DOC 49 kb)
