# Supplementary material for: Improving appropriate polypharmacy for older people in primary care: selecting components of an evidence-based intervention to target prescribing and dispensing
Source: Implement Sci. 2015 Nov 16;10:161. doi: 10.1186/s13012-015-0349-3 (PMC4647274; doi:10.1186/s13012-015-0349-3)
Supplement: Additional file 1: — General practitioner interview topic guide. TDF-based topic guide that was used to explore the theoretical domains as barriers and facilitators to the prescribing of appropriate polypharmacy for older people.(DOC 62 kb) [file 13012_2015_349_MOESM1_ESM.doc]

### Additional file 1 General practitioner interview topic guide

# GP interview schedule

## Introduction

“Thank you very much for making the time to talk to me today.

Have you had a chance to read through the information sheet that was sent out to you?

So the aim of this interview is to explore your views of polypharmacy in older people, your approach to prescribing polypharmacy for this age group and your perception of the barriers and facilitators to achieving appropriate polypharmacy for older patients in primary care. I’d like to focus specifically on older patients living within the community as opposed to those in nursing home or residential care home settings.

The interview should last [estimated duration] minutes.

Before we start I just need to get written consent from you that you understand what the study involves; that you know that anything you say will be kept completely confidential; that you will not be identified in any way; that you know that we can stop at any time; that you are happy for the interview to be recorded. If you wouldn’t mind, can you read through this consent form and initial each box to indicate that you understand and agree with each statement?

[Turn recorder on]

Have you any immediate questions about the study before we get started on the interview?”

## A. Demographics

- Could you tell me how long you have been practising as a GP?
- Approximately, what percentage of the patients in this practice are older patients (by older I mean ≥65 years)?
- On a typical working day in your practice:
  - Approximately what percentage of your overall prescribing is for older patients?
  - What would be the average number of items regularly prescribed per older patient?

## Definitions

How would you define polypharmacy?

**PROMPTS** (depending on response):

- Can you tell to me why you find it difficult to define polypharmacy?
- Can you tell me how you came to define polypharmacy as X number of medicines?

There are several definitions of polypharmacy in the literature. For the purpose of this project, we are adopting a definition of polypharmacy which states that…

[Hand participant printed flashcard of definitions]

- **Polypharmacy** constitutes the co-prescribing of four or more regular medicines (Cochrane Review)
- **Appropriate polypharmacy** is defined as prescribing for an individual for complex conditions or for multiple conditions in circumstances where medicines use has been optimised and where the medicines are prescribed according to best evidence.

However, as you can see from these definitions others have classified it differently. This whole concept of appropriate polypharmacy is really about recognising that some patients may benefit from multiple medicines and highlighting the importance of getting the balance right between many and too many drugs.

Obviously this can be a difficult judgement call but that’s why we need to talk to you about what makes it clear, unclear, difficult, easy, etc. Is that ok?

## B. Behavioural elicitation

Before we talk about the issues around the prescribing of appropriate polypharmacy in older people, could you describe your approach to issuing a prescription for multiple medications to a typical older patient in your practice?

**Prompt**: How would you start the prescribing process for an older person?

Potential generic prompts here (if appropriate)–

- What would you do next?
- Anything else?
- [possibly] Would you always do these things in the same order?
- Can you think of any exceptions to this pattern?

## C. Polypharmacy scenarios

I’m now going to show you an example of inappropriate polypharmacy. This scenario is not intended as a test but I would be interested to get your opinion on issues of potential concern with the patient’s medication list. I would like you to take a few minutes to review the patient’s medication list. When you’re ready, I’d like to ask you about your main concerns with the medications being prescribed and the key issues that you would prioritise if you were to attempt to make any changes to the patient’s prescription. Does that sound ok to you?

Take your time to have a look through this list and when you’re ready I will ask you some questions.

[Hand participant printed flashcard of clinical scenario]

***Give interviewee a few minutes to review ***

Mr John Moore is a 72 year old retired taxi driver. He lives in sheltered accommodation with his wife who suffers from depression. Over time he has become less mobile and is now unable to visit the surgery. He requests regular house calls from his general practitioner.

Over the last 6 months, he has received a prescription for all of the repeat medications at the start of every month.

There have been no changes or records of any communication between the GP and community pharmacist in the patient’s notes in the last six months.

**PAST MEDICAL HISTORY**

- Fibromyalgia Syndrome
- General anxiety disorder
- Depression
- COPD (moderate)
- Headaches
- Falls
- Chronic kidney disease (Stage 3)
- Hypertension
- Benign Prostatic hypertrophy
- Diverticular disease
- Knee and hip osteoarthritis
- Obesity (BMI 36)

**SOCIAL HISTORY**

Smoker 20 cigarettes per day over the last 50 years.

| **INAPPROPRIATE POLYPHARMACY** |
| --- |
| **ACUTE MEDICATION (in last 2 months)** |
| TEMAZEPAM 10mg one nocte (28) |
| DICLOFENAC gel (1% w/w) one application TDS PRN (100g) |
| TEMAZEPAM 10mg nocte (28) |
| CHLORPHENIRAMINE 4mg one BD PRN (56) |
|  |
| **REPEAT MEDICATION** |
| LACTULOSE 10mls BD PRN (300mls) |
| MACROGOL one-two sachets mane PRN (60) |
| TRAMADOL MR 100mg 1 BD (56) |
| CO-CODAMOL 30/500 mg one or two 4-6 HOURLY PRN Max 8 per day (100) |
| AMITRIPTYLINE 50mg NOCTE (28) |
| SALBUTAMOL one – two puffs PRN (4) |
| CLENIL MODULITE (beclometasone dipropionate) 100mcg one puff BD (1) |
| IBUPROFEN 400mg one 8 hourly |
| GLUCOSAMINE 1500mg one daily (28) |
| CITALOPRAM 20mg one daily (28) |
| DIAZEPAM 2mg ONE BD PRN (56) |
| BENDROFLUMETHIAZIDE 2.5mg one mane (28) |
| TAMSULOSIN 0.4 mg SR one daily (28) |
| SLOW SODIUM (sodium chloride 600 mg)two daily (56) |

I know that we could potentially spend the rest of the interview discussing this scenario alone, but from your initial review of this patient’s record, can you tell me briefly what the main issues are that you would be concerned about?

If you were to attempt to address these issues, what would you prioritise and why?

*Issues for Cathal to be aware of in case referred to by interviewee*

- *receiving diazepam every month and has also recently been receiving temazepam.*
- *receiving two anti-depressants: a TCA and SSRI*
- *receiving both oral and topical NSAIDs, as well as two opioids*
- *Hypertension: receiving bendroflumethiazide…not first-line treatment of choice for hypertension, implications in terms of sodium loss (especially combined with SSRI) and need for sodium supplementation.*
- *Laxatives: duplication*
- *Inhalers: use long-acting beta agonist instead of a short-acting*
- *Glucosamine: GPs have actively been discouraged from prescribing*

I’d now like to ask you some general questions around potential barriers and facilitators to achieving appropriate polypharmacy in this patient, again there are no right or wrong answers.

### *Knowledge*

**Q.** What knowledge do you have as a GP that would help you to make the necessary changes to this patient’s prescription to ensure that he is receiving appropriate polypharmacy as opposed to inappropriate polypharmacy?

**Prompt**: Clinical knowledge?

- - - Specific knowledge sources/resources?
    - Anything specifically relating to polypharmacy in older people?

**Prompt**: Knowledge of guidelines?

- - - What guidelines?
    - What would the guidelines recommend?

**Q.** Assuming that you had the time, what sources of information would you check to help you to make the necessary changes to this patient’s prescription?

*Skills*

You identified a number of issues of concern with the medications listed in the patient’s record, such as [cite issues e.g. benzodiazepines, antidepressants, NSAIDs].

**Q.** What skills do you have as a GP to address these issues?

**Prompt:** What skills do you have that would help you to engage with the patient?

**Prompt:** How might that skill play out in the scenario?

**Prompt:** What skills do you have that would help you to engage with other healthcare professionals?

**Q.** Going forward, what training would be helpful to you in addressing the issues that you identified effectively?

### *Social/professional role and identity*

**Q.** Thinking about polypharmacy in older people, what would you consider your responsibilities to be as a GP in ensuring that older patients receive appropriate polypharmacy)?

**Prompt:** Is there anything that you would consider to be beyond your responsibility as a GP (in ensuring that patients receive appropriate polypharmacy)?

### *Beliefs about capabilities*

**Q.** Could you tell me about your confidence in identifying inappropriate polypharmacy and making the necessary changes, such as those which you identified in Mr. Moore’s medical record?

**Prompt:** In general, in what situations would you be confident to make these changes?

**Prompt:** And can you describe to me any situations where you would not feel so confident in making these changes?

### *Beliefs about consequences*

**Q.** Focussing on the example of Mr Moore, what do you think are the benefits of addressing the issues that you identified with the medications that are being prescribed to him?

**Q.** Can you think of any downsides that would be associated with making these changes to the medications that are being prescribed to him?

**Prompt:** What impact would this have on your decision to take action?

### *Motivation and goals*

**Q.** How important is it to **you** to try and change the medications that he is being prescribed?

**Q.** In what circumstances would you think it was less important to make these changes?

### *Memory, attention and decision processes*

**Q.** Can you talk me through how you would decide to take the necessary steps to resolve the issues with the medications he is being prescribed?

**Q.** Are there any circumstances in which you might just forget to make these changes?

### *Behavioural regulation*

**Q.** Having decided the best course of action from a clinical point of view, are there any circumstances which might prevent you from taking this action?

**Q.** Are there any strategies you would use to overcome these circumstances?

### *Nature of the behaviours*

**Q.** Is there anything that you do routinely in your everyday practice to ensure that you prescribe appropriately for older patients who need four or more drugs, such as in the case of Mr. Moore?

### *Environmental context and resources*

**Q.** What resources might help you to intervene when you encounter older patients receiving inappropriate polypharmacy?

**Q.** Are there any aspects of your work environment that might prevent you from addressing the issues with the medications that Mr Moore is being prescribed?

### *Social influences*

**Q.** Who would influence your decisions about whether to change the medicines on the prescription?

Generic prompts: Individuals/groups/agencies? Anyone else?

**Prompt**: Can you tell me more about how that happens?

### *Emotion*

**Q.** If this patient was to become upset or anxious as a result of your attempts to resolve the issues with the medications being prescribed to him, how would this influence your decision to take action?

**Q.** How does work stress influence your decision influence your decision to attempt to resolve the issues with this prescription?

## D. Intervention components

Our research group is interested in interventions/approaches to support appropriate polypharmacy in older patients. From reviewing the literature we have found that interventions can be quite complex and often involve a number of different components. This makes it difficult to identify which components are the most important in terms of improving patient outcomes and achieving appropriate polypharmacy.

Just to give you an idea of what has previously been done…

- Some interventions have had an educational focus, such as providing prescribers with evidence-based information or educating patients about their medicines.
- In other cases pharmacists have conducted independent medication reviews and made recommendations to patients’ prescribers as part of a multidisciplinary team
- One study used what’s known as computerised decision support to alert prescribers to clinically relevant prescribing problems in patients’ records; the alerts identified the nature of the problem, possible consequence and suggested alternative therapy.

**Q.** What would you consider to be important components of an intervention to improve appropriate polypharmacy for older people in primary care settings?

**Prompt:** Who should be involved in delivering these types of interventions in practice? (pharmacists, carer?, GP, practice nurse)

**Prompt:** What would each person/health-care practitioner have to do?

**Prompt:** What are your thoughts on patient involvement in interventions -should patients be actively involved in the decisions about the medicines they are prescribed?

**Q.** What would the barriers be to putting the type of intervention that you have described into practice?

**Q.** What would help the implementation of the intervention?

**Q.** What do you think should be measured as an outcome in an intervention study to support appropriate polypharmacy i.e. how would you, personally, be persuaded that the intervention had improved appropriate polypharmacy? What are the most important ones?

## Concluding comments

That brings us to the end of the interview.

Is there anything else about the topic of appropriate polypharmacy in older people that you feel has not been covered?

Do you have any additional comments that you would like to make as to the content of the interview or how it went?

Thank you very much for giving up your time to talk to me today.
