# Supplementary material for: Improving appropriate polypharmacy for older people in primary care: selecting components of an evidence-based intervention to target prescribing and dispensing
Source: Implement Sci. 2015 Nov 16;10:161. doi: 10.1186/s13012-015-0349-3 (PMC4647274; doi:10.1186/s13012-015-0349-3)
Supplement: Additional file 2: — Community pharmacist interview topic guide. TDF-based topic guide that was used to explore the theoretical domains as barriers and facilitators to the dispensing of appropriate polypharmacy to older people. (DOC 58 kb) [file 13012_2015_349_MOESM2_ESM.doc]

### Additional file 2 Community pharmacist interview topic guide

# Community pharmacist interview schedule

## Introduction

Thanks very much for making the time to talk to me today.

Have you had a chance to read through the information sheet that was sent out to you?

So the aim....of this interview is to explore your views of polypharmacy in older people, your approach to **dispensing polypharmacy** for this age group and your perception of the barriers and facilitators to achieving appropriate polypharmacy for older patients in primary care.

The interview should last [estimated duration] minutes.

“Before we start I just need to get written consent from you that you understand what the study involves, that you know that anything you say will be kept completely confidential and you will not be identified in any way, you know that we can stop at any time and are happy for the interview to be recorded? If you wouldn’t mind reading through this consent form and initialling each box to indicate that you understand and agree with each statement.

Have you any immediate questions about the study before we get started on the interview?”

## A. Demographics

- Could you tell me how long you have been practising as a community pharmacist?
- Approximately, what percentage of the patients in this pharmacy are older patients (by older I mean ≥65 years)?
- On a typical working day in your pharmacy:
  - Approximately what percentage of your overall dispensing activities is for older patients?
  - Average number of dispensed items per older patient?
  - *What* percentage *would you say are repeat items?*

## Definitions

I’d now like to ask you about the term polypharmacy itself….

How would you define polypharmacy?

**PROMPT**: Can you tell to me why you find it difficult to define polypharmacy?

**PROMPT**: Can you tell me how you came to define polypharmacy as X number of medicines?

There are several definitions of polypharmacy in the literature. For the purpose of this project, we are adopting a definition of polypharmacy which states that…

[Hand participant printed flashcard of definitions]

- **Polypharmacy** constitutes the co-prescribing of four or more regular medicines (Cochrane Review)
- **Appropriate polypharmacy** is defined as prescribing for an individual for complex conditions or for multiple conditions in circumstances where medicines use has been optimised and where the medicines are prescribed according to best evidence.

So it’s about getting the balance right between many and too many drugs.

Obviously this is sometimes a difficult judgement call but that’s why we need to talk to you about what makes it clear, unclear, difficult, easy, etc. Is that ok?

## B. Behavioural elicitation

Before we talk about the issues around the dispensing of appropriate polypharmacy in older people, could you describe your approach to dispensing a prescription for multiple medications to a typical older patient in your pharmacy?

**Prompt**: How would you start the dispensing process?

Potential generic prompts here (if appropriate)–

- What would you do next?
- Anything else?
- [possibly] Would you always do these things in the same order?
- Can you think of any exceptions to this pattern?

## C. Polypharmacy scenarios

I’m now going to show you an example of an older patient receiving inappropriate polypharmacy. This scenario is not intended as a test. I would like for you to take a few minutes to review the patient’s medication list. When you’re ready I’d like to ask you a few questions about your **main concerns** with the prescription and the **key issues that you would prioritise** if you were to attempt to make any changes to the patient’s prescription. Does that sound ok to you? Take your time to have a look through this list and when you’re ready I will ask you some questions.

I would then like to ask you some general questions around potential barriers and facilitators to achieving appropriate polypharmacy in this patient.

[Hand participant printed flashcard of clinical scenario]

The patient, Mr. Moore, is a 72 year old male who typically receives 14 prescription medicines each month on a repeat prescription. Based on the patient’s medication record, he has had all of the repeat medications on this prescription dispensed in his local pharmacy for the last 6 months. There have been no changes or records of any communication between the GP and community pharmacist.

Please take a few minutes to have a look over this patient’s prescription record.

***Give interviewee a few minutes to review ***

| **INAPPROPRIATE POLYPHARMACY** |
| --- |
| **ACUTE MEDICATION (in last 2 months)** |
| TEMAZEPAM 10mg one nocte (28) |
| DICLOFENAC gel one application TDS PRN (100g) |
| TEMAZEPAM 10mg nocte (28) |
| CHLORPHENIRAMINE 4mg one BD PRN (56) |
|  |
| **REPEAT MEDICATION** |
| LACTULOSE 10mls BD PRN (300mls) |
| MACROGOL one-two sachets mane PRN (60) |
| TRAMADOL MR 100mg 1 BD (56) |
| CO-CODAMOL 30/500 one or two 4-6 HOURLY PRN Max 8 per day (100) |
| AMITRIPTYLINE 50mcg NOCTE (28) |
| SALBUTAMOL one – two puffs PRN (4) |
| CLENIL MODULITE 100mcg one puff BD (1) |
| IBUPROFEN 400mg one 8 hourly |
| GLUCOSAMINE 1500mg one daily (28) |
| CITALOPRAM 20mg one daily (28) |
| DIAZEPAM 2mg ONE BD PRN (56) |
| BENDROFLUMETHIAZIDE 2.5mg one mane (28) |
| TAMSULOSIN SR 400 mcg one daily (28) |
| SLOW SODIUM two daily (56) |

Ok I know that we could potentially spend the rest of the interview discussing this scenario alone, instead though, just based on your initial review **could you tell me briefly what are the main issues that you would be concerned** about**?**

If you were to attempt to **address these issues**, what would you **prioritise and why**?

Potential generic prompts, if appropriate (ie more behavioural elicitation):

- How would you go about addressing this?
- Who would you contact first?
- What, if anything, would you say to the patient?

*Issues for Cathal to be aware of in case referred to by interviewee*

- *receiving diazepam every month and has also recently been receiving temazepam.*
- *receiving two anti-depressants: a TCA and SSRI*
- *receiving both oral and topical NSAIDs, as well as two opioids*
- *Hypertension: receiving bendroflumethiazide…not first-line treatment of choice for hypertension, implications in terms of sodium loss (especially combined with SSRI) and need for sodium supplementation.*
- *Laxatives: duplication*
- *Inhalers: use long-acting beta agonist instead of a short-acting*

I’m going to ask you a few questions based on this scenario, again there are no right or wrong answers.

### *Knowledge*

**Q.** What **knowledge** do you have as a pharmacist that would help you to **make the necessary changes** to this patient’s prescription to ensure that he is receiving appropriate polypharmacy as opposed to inappropriate polypharmacy?

**Prompt**: Clinical knowledge?

- - - Specific knowledge sources/resources?
    - Anything specifically relating to polypharmacy in older people?

**Prompt**: Knowledge of guidelines?

- - - What guidelines?
    - What would the guidelines recommend?

**Q.** Assuming that you had the time, **what sources of information would you check to** help you to make the necessary changes to this patient’s prescription?

*Skills*

You identified a number of issues of concern with this patient’s prescription, such as [cite issues e.g. benzodiazepines, antidepressants, NSAIDs].

**Q.** What skills do you have as a pharmacist to address these issues?

**Prompt:** What skills do you have that would help you to engage with the patient?

**Prompt:** What skills do you have that would help you to engage with the prescriber?

**Q.** Going forward, what training would be helpful to you in addressing these issues effectively?

### *Beliefs about capabilities*

**Q.** Could you tell me about your **confidence in identifying inappropriate polypharmacy and making the necessary changes**, such as those which you identified in the case of Mr. Moore’s prescription?

**Prompt:** In general, in what situations would you be confident to make these changes?

**Prompt:** And can you describe to me any situations where you would not feel so confident in making these changes?

### *Social/professional role and identity*

**Q.** Thinking about polypharmacy in older people, **what would you consider your responsibilities to be within the multidisciplinary team** (in ensuring that older patients receive appropriate polypharmacy)?

**Prompt:** Is there anything that you would consider to be beyond your responsibility as a pharmacist (in ensuring that patients receive appropriate polypharmacy)?

### *Beliefs about consequences*

**Q.** Focussing on the example of Mr Moore, what do you think are the **benefits of addressing the issues** that you identified with his prescription?

**Q.** Can you think of any downsides that would be associated with making these changes to his prescription?

### *Motivation and goals*

**Q.** How **important** is it to you to try and change this prescription?

**Q.** In **what circumstances would you think it was less important** to make these changes?

### *Nature of the behaviours*

**Q.** Is there **anything that you do routinely** in your everyday practice to resolve the issues with this kind of prescription?

### *Memory, attention and decision processes*

**Q.** Can you **talk me through how you would decide** to take the necessary steps to resolve the issues with this prescription?

**Q.** Are there any circumstances in which you might just forget to make these changes?

### *Social influences*

**Q. Who would influence your decisions** about whether to recommend changes to the medicines on the prescription?

Generic prompts: Anyone else?

**Prompt**: Can you tell me more about how that happens?

### *Emotion*

**Q.** If this patient was **upset or anxious because of your attempts** to attempt to resolve the issues with his prescription**, how would this influence your decision to proceed further and seek to implement change**?

**Q.** How does work stress might influence your decision to attempt to resolve the issues with this prescription?

### *Behavioural regulation*

**Q.** How would you go about making the necessary changes to this patient’s prescription?

**Prompt**: What are the practical steps/strategies that you could put in place to ensure that the medication that is dispensed to the patient is appropriate for him?

**Q.** What would stop you putting those practical steps/strategies into place?

**Q.** What would make it easier to do so?

### *Environmental context and resources*

**Q.** What **resources** might help you to intervene when inappropriate prescriptions are presented to you?

**Q.** Are there any **aspects of your work environment** that prevent you from addressing the issues with this patient’s prescription prior to dispensing?

## D. Intervention components

Our research group is interested in interventions or approaches to support appropriate polypharmacy in older patients. From reviewing the literature we have found that interventions can be quite **complex** and often involve a **number of different components**. This makes it **difficult to identify which components are the most important** in terms of improving patient outcomes and achieving appropriate polypharmacy.

Just to give you an idea of what has previously been done…

- In some cases **pharmacists** have conducted **independent medication reviews** and made recommendations to patients’ prescribers as part of a **multidisciplinary team**
- Other interventions have had more of an **educational focus**, such as providing prescribers with evidence-based information or educating patients about their medicines.
- One study used what’s known as **computerised decision support** to alert prescribers to clinically relevant prescribing problems in patients’ records; the alerts identified the nature of the problem, possible consequence and suggested alternative therapy.

**Q.** What would you consider to be **important components** of an intervention to improve appropriate polypharmacy for older people in primary care settings?

**Prompt:** Who should be involved in delivering these types of interventions in practice? (pharmacists, carer?, GP, practice nurse)

**Prompt:** What would each person/health-care practitioner have to do?

**Prompt:** What are your thoughts on patient involvement in interventions -should patients be actively involved in the decisions about the medicines they are prescribed?

**Q.** What would the **barriers** be to putting the type of intervention that you have described into practice?

**Q.** What would help the implementation of the intervention?

The effect of every intervention should be measured.

**Q.** What do you think should be measured as an outcome in an intervention study to support appropriate polypharmacy i.e. how would you, personally, be persuaded that the intervention had improved appropriate polypharmacy? What are the most important ones?

## Concluding comments

That brings us to the end of the interview.

Is there anything else about the topic of appropriate polypharmacy in older people that you feel has not been covered?

Do you have any additional comments that you would like to make as to the content of the interview or how it went?

Thank you very much for giving up your time to talk to me today.
