# Supplementary material for: Improving appropriate polypharmacy for older people in primary care: selecting components of an evidence-based intervention to target prescribing and dispensing
Source: Implement Sci. 2015 Nov 16;10:161. doi: 10.1186/s13012-015-0349-3 (PMC4647274; doi:10.1186/s13012-015-0349-3)
Supplement: Additional file 3: — Description of 12 theoretical domains from TDF [ 13 ]. Descriptions of the theoretical domains that were used to code the interview data. (DOC 49 kb) [file 13012_2015_349_MOESM3_ESM.doc]

### Additional file 3: Description of 12 theoretical domains from TDF [13]

| **Domain label** | **Description of domain content** |
| --- | --- |
| Knowledge | Knowledge of the field (i.e. whether there is adequate evidence) and individuals’ knowledge of the evidence or of a guideline. |
| Skills | Covers the possibility that new skills would be required by the staff who are required to implement a new procedure. |
| Social/professional role and identity | The clinical thinking and norms of a particular profession. |
| Beliefs about capabilities | How confident clinicians are that they could change their practice effectively |
| Beliefs about consequences | Often regarded as core to clinical reasoning, this domain covers the perceived benefits and harms of a clinical action. In some contexts it can also include consequences for the clinician such as workload, pay, career progression, or for the hospital or health service. |
| Motivation and goals | The relative priority that is given to one clinical issue, compared with other demands. |
| Memory, attention and decision processes | The level of attention that is needed to perform the key clinical action (i.e. is forgetting likely to be a problem) and the processes by which clinical decisions are made by individuals and teams. |
| Environmental context and resources | Includes the physical (including financial) issues that may limit change, including staffing levels and time as well as equipment or space. |
| Social influences | The influence of other individuals or groups on clinical practice, for example, patients, patients’ families, pressure groups. |
| Emotion | Includes issues such as work stress, patient anxiety and other emotional factors that may help or hinder the uptake of new approaches to care. |
| Behavioural regulation | Includes the ‘how’ of changing clinical practice: what are the practical strategies that would facilitate or hinder uptake of a new practice. |
| Nature of the behaviours | Some new practices are very similar to current practice and so are easier to implement than new practices that require a dramatic change in ways of working. |

Source: Cuthbertson *et al.* [28]
